# Lactate-Induced Dispersal of *Neisseria meningitidis* Microcolonies Is Mediated by Changes in Cell Density and Pilus Retraction and Is Influenced by Temperature Change

**DOI:** 10.1128/IAI.00296-21

**Published:** 2021-09-16

**Authors:** Sara Sigurlásdóttir, Kenny Lidberg, Fanglei Zuo, Jane Newcombe, Johnjoe McFadden, Ann-Beth Jonsson

**Affiliations:** a Department of Molecular Biosciences, The Wenner-Gren Institute, Stockholm University, Stockholm, Sweden; b School of Biosciences and Medicine, Faculty of Health and Medical Sciences, University of Surrey, Guildford, United Kingdom; University of California—San Diego School of Medicine

**Keywords:** *Neisseria meningitidis*, lactate, microcolony dispersal

## Abstract

Neisseria meningitidis is the etiologic agent of meningococcal meningitis and sepsis. Initial colonization of meningococci in the upper respiratory tract epithelium is crucial for disease development. The colonization occurs in several steps and expression of type IV pili (Tfp) is essential for both attachment and microcolony formation of encapsulated bacteria. Previously, we have shown that host-derived lactate induces synchronized dispersal of meningococcal microcolonies. In this study, we demonstrated that lactate-induced dispersal is dependent on bacterial concentration but not on the quorum-sensing system autoinducer-2 or the two-component systems NarP/NarQ, PilR/PilS, NtrY/NtrX, and MisR/MisS. Further, there were no changes in expression of genes related to assembly, elongation, retraction, and modification of Tfp throughout the time course of lactate induction. By using *pilT* and *pptB* mutants, however, we found that lactate-induced dispersal was dependent on PilT retraction but not on phosphoglycerol modification of Tfp even though the PptB activity was important for preventing reaggregation postdispersal. Furthermore, protein synthesis was required for lactate-induced dispersal. Finally, we found that at a lower temperature, lactate-induced dispersal was delayed and unsynchronized, and bacteria reformed microcolonies. We conclude that lactate-induced microcolony dispersal is dependent on bacterial concentration, PilT-dependent Tfp retraction, and protein synthesis and is influenced by environmental temperature.

## INTRODUCTION

Bacterial pathogens encounter constant changes in their environment and must respond quickly to survive and proliferate (1). Neisseria meningitidis is a human-restricted pathogen that is well adapted to survival in the upper respiratory tract. The nasopharyngeal epithelium is the natural reservoir for meningococci; however, bacteria can penetrate the cell barrier and cause sepsis and/or meningitis (2). Initial colonization of the mucosal epithelium is crucial for disease development and is mediated by type IV pili (Tfp). Once Tfp-mediated attachment to the mucosa has been established, meningococci start forming microcolonies. Microcolony formation on host cells stimulates cytoskeletal rearrangement that can contribute to bacterial resistance against physical forces (3). However, to invade the mucosa, bacteria must disperse from the microcolonies and form close contacts with host cells (4–6).

Several factors have been demonstrated to contribute to the dispersal of microcolonies. The ATPase PilT is responsible for Tfp retraction. A *pilT* deletion mutant exhibited a hyperpiliated aggregative phenotype unable to disperse and form intimate adhesion to host cells (5). It has been proposed that an initial contact with host cells results in increased expression of *pptB* encoding pilin phosphotransferase B. Upregulation of *pptB* results in posttranslational modifications (PTM) by adding phosphoglycerol to PilE, which blocks the bundle formation and leads to dispersal (6). Environmental oxygen concentration has been demonstrated to be important for microcolony formation and dispersal in pathogenic *Neisseria*. At low oxygen concentrations, the loss of the proton motive force leads to microcolony instability and dispersal (7). Recently, we demonstrated that a host-derived metabolite, lactate, can induce microcolony dispersal (8). Lactate is known to promote neisserial metabolic rate and oxygen consumption (9, 10). However, the lactate-induced dispersal did not depend on metabolic utilization and reduced oxygen concentration, suggesting lactate as a signaling cue (8). In addition, we demonstrated that lactate was unable to prevent microcolony formation (8). This suggests that another mechanism must be involved in order to sense lactate. The bacteria had to form microcolonies in order to respond to the metabolite, indicating the involvement of additional factors.

Quorum sensing (QS) plays an important role in bacterial behavior in a density-dependent manner. The role of QS in *Neisseria* is poorly understood. It is known that meningococcal LuxS produces the QS molecule autoinducer-2 (AI-2) (11, 12). However, the presence of a *luxP* homologue, encoding the AI-2 receptor protein has not yet been detected, and meningococci have been shown to be nonresponsive to AI-2 (11, 13). Deletion of *luxS* in meningococci has been reported to affect virulence in a rat model (12). Later, it was speculated that accretion of toxin metabolic intermediate due to deletion of *luxS* was the reason for the observed phenotype (14).

Another way for bacteria to sense and adapt to external signals is to use two-component systems (TCS). Pathogenic *Neisseria* is known to encode only four TCS (15). The MisR/MisS system is activated upon host cell contact and regulates the expression of genes involved in adaptation to growth on host cells (16–18). The NarP/NarQ and NtrX/NtrY systems control the expression of genes involved in denitrification, thus promoting anaerobic survival. (19–22). Finally, the meningococci also encode the PilR/PilS system. In Pseudomonas aeruginosa, the PilR/PilS system is known to control piliation (23). A system resembling the PilR/PilS exists in *Neisseria*, but it was shown not to affect piliation (24).

N. meningitidis can experience fluctuations in temperature during its colonization. It is transmitted through aerosol droplets to the human nasopharynx with an environmental temperature that can alternate between 32 and 34°C (25, 26). Changes in the environmental temperature can affect the virulence of N. meningitidis. A recent study has shown that when N. meningitidis is grown at 32°C, this increases aggregation, adherence to host cells, and biofilm formation compared to processes at 37°C, and the protein levels of virulence factors involved in this process are increased (27). In addition, meningococci use RNA thermosensors to detect temperature changes, which can influence the expression of virulence factors (28, 29).

Here, we sought to understand the mechanism behind lactate-induced microcolony dispersal in N. meningitidis. We demonstrated that the dispersal required protein synthesis and occurred in a density-dependent manner, although the QS system autoinducer AI-2 was not involved. Further, deletion of each of the four TCS had no effect on microcolony dispersal. Deletion of *pilT* blocked dispersal, suggesting its dependence on Tfp retraction. The phosphoglycerol modification by PptB was required for bacteria to remain dispersed but not for dispersal itself. Finally, we found that dispersed bacteria reformed microcolonies at 32°C but not at 37°C, suggesting that a temperature-dependent mechanism is involved.

## RESULTS

### Initiation of *N. meningitidis* lactate-induced microcolony dispersal is dependent on bacterial concentration but not on AI-2 production.

**FIG 1:**
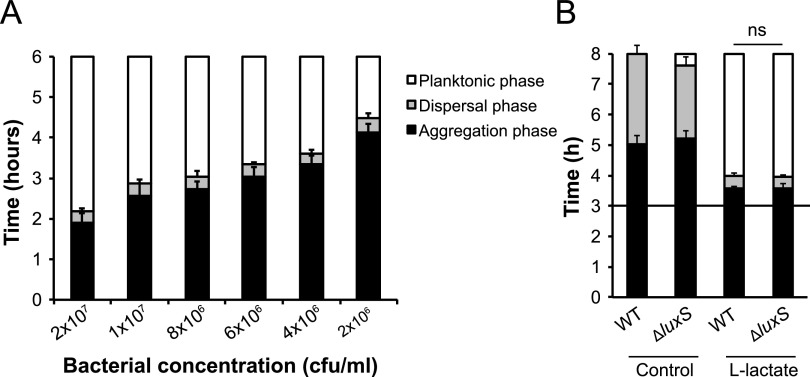
Initiation of N. meningitidis lactate-induced microcolony dispersal is dependent on bacterial concentration but not on AI-2 production. (A) Bacteria were resuspended in medium with of 2 mM l-lactate, filtered through a 5-μm filter to break preexisting aggregates, and diluted to suspensions with different concentrations ranging from 2 × 10^6^ to 2 × 10^7^ CFU/ml. The timing of microcolony dispersal was examined by live-cell imaging. (B) Induction assays comparing microcolony dispersal of the wild-type (WT) and Δ*luxS* bacteria. Bacteria were resuspended to 10^7^ CFU/ml in DMEM containing 1% FBS and allowed to form aggregates for 3 h. Lactate solutions were added to the final concentrations of 10 mM at a 1:1 ratio, and microcolony dispersal was followed by live-cell imaging. A black horizontal line represents the 3-h time point of induction in panel B. DMEM was added as a control. The bars represent the means of three independent experiments. Error bars represent the standard deviations. ns, nonsignificant.

We have previously shown that host-derived lactate induces fast and synchronized microcolony dispersal in N. meningitidis (8). We found that lactate, although required for microcolony dispersal, did not prevent microcolony formation. When bacteria were inoculated in growth medium (10^7^ CFU/ml) with 2 mM lactate, it took 2 h to form microcolonies and, after an additional 30 min, the microcolonies dispersed in a synchronized way (8). We therefore examined whether the lactate-mediated dispersal was dependent on cell density. First, we incubated bacteria at different concentrations in the presence of lactate and followed the timing of dispersal by live-cell microscopy. As shown in Fig. 1A, the initiation of microcolony dispersal was dependent on bacterial concentration; the more bacteria were present in the suspension, the earlier the dispersal occurred. Consequently, we speculated that a density-dependent mechanism such as QS might trigger dispersal. To examine the role of quorum sensing, we constructed a *luxS*-deficient mutant (Δ*luxS*). However, the Δ*luxS* mutant showed no difference in timing of dispersal from that of the wild-type strain (Fig. 1B). Taken together, these results show that N. meningitidis lactate-induced dispersal occurs in a density-dependent manner; however, it is not affected by disruption of AI-2 production.

### The TCS PilR/PilS, NarP/NarQ, NtrY/NtrX, and MisR/MisS are not required for *N. meningitidis* lactate-induced dispersal.

**FIG 2:**
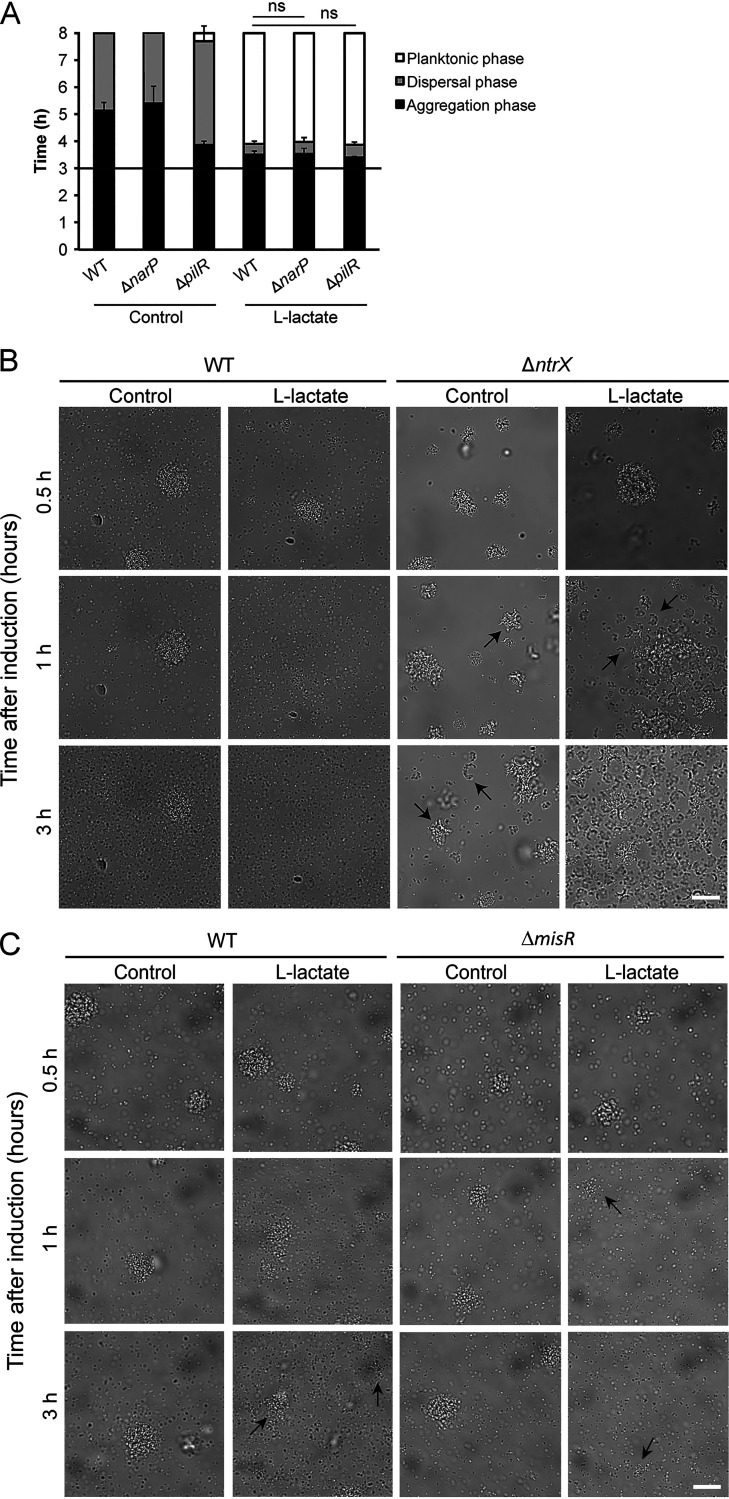
The TCS PilR/PilS, NarP/NarQ, NtrX/NtrY, and MisR/MisS are not involved in N. meningitidis microcolony dispersal. (A) Induction assays comparing microcolony dispersal of wild-type (WT), Δ*narP*, and Δ*pilR* bacterial strains. Bacteria were resuspended to 10^7^ CFU/ml in DMEM containing 1% FBS and allowed to form aggregates for 3 h. Lactate solutions were added to the final concentrations of 10 mM at a 1:1 ratio, and microcolony dispersal was followed by live-cell imaging. A black horizontal line represents the 3-h time point of induction. DMEM was added as a control. The bars represent the means of three independent experiments. Error bars represent the standard deviations. ns, nonsignificant. (B) Microscopy images showing FAM20 wild-type (WT) and Δ*ntrX* strains upon the addition of 10 mM l-lactate. Black arrows mark examples of abnormal bacterial morphologies. DMEM was added as a control. Representative images are shown for 0.5, 1, and 3 h. Scale bar, 20 μm. (C) Microscopy images showing M96255789 wild-type (WT) and Δ*misR* strains upon the addition of 10 mM l-lactate. Black arrows mark minor remaining microcolonies. DMEM was added as a control. Representative images are shown for 0.5 h, 1 h, and 3 h. Scale bar, 20 μm.

To further identify the potential signaling behind the lactate-induced dispersal, we examined the role of TCS. In Sigurlásdóttir et al. (8), we found no difference in the mRNA level of the TCS-regulator MisR upon lactate-induced dispersal. However, not only the amount of the response regulator but also the phosphorylation level can affect the genes controlled by the systems (30). We therefore attempted to generate deletion mutants of all the TCS regulators encoded by N. meningitidis FAM20 to examine their involvement. We successfully generated Δ*narP* (regulator of NarP/NarQ), Δ*pilR* (regulator of PilR/PilS), and Δ*ntrX* (regulator of NtrY/NtrX) deletion mutants in strain FAM20 and confirmed them by PCR. Despite several attempts, we were unable to create a FAM20 Δ*misR* (regulator of MisR/MisS) deletion mutant, indicating heterogeneity among strains and a possible essential role of MisR in FAM20. Therefore, we used an already existing Δ*misR* mutant in the serogroup C isolate N. meningitidis L91543 (31). In induction assays, we observed that deletion of *narP* and *pilR* did not affect the timing of dispersal in the presence of lactate (Fig. 2A). Although we observed dispersal of Δ*ntrX* aggregates upon addition of l-lactate, we noted that Δ*ntrX* mutant gave rise to unusual cell morphology in live-cell images, suggesting that Δ*ntrX* mutant might suffer abnormalities during cell division (Fig. 2B). We also observed dispersal of both L91543 wild-type and Δ*misR* (previous known as Δ*phoP* [31]) aggregates upon addition of l-lactate. However, the dispersal duration was longer than that of FAM20 and the microcolonies could not completely disperse (Fig. 2C). In summary, the data suggest that regulators of NarP/NarQ, PilR/PilS, NtrX/NtrY, and MisR/MisS TCS are not involved in lactate-induced N. meningitidis microcolony dispersal.

### *N. meningitidis* lactate-induced microcolony dispersal requires PilT retraction and PptB to remain dispersed.

Pilus-associated factors are well known to affect clumping and aggregation of the bacteria. We next investigated the transcripts of genes related to assembly (*pilE*, *pilD*, and *pilQ*), elongation (*pilF*), retraction (*pilT*), and modification (*pptB*) of Tfp throughout the time course of lactate induction. However, no significant changes in gene expression were detected at 1, 5, 10, 20, 30, or 60 min after lactate addition (see Fig. S1 in the supplemental material).

**FIG 3:**
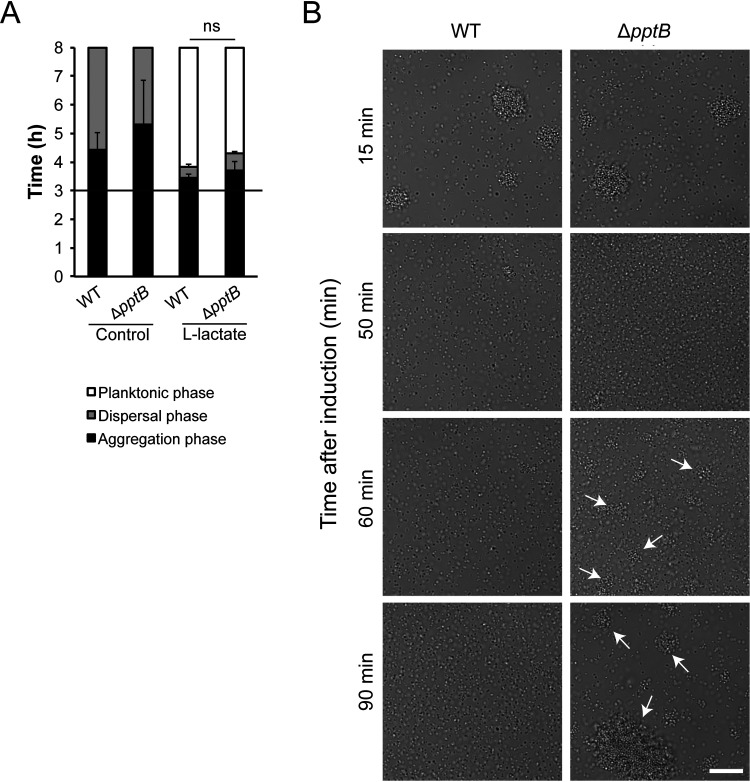
Meningococci depend on PptB to remain dispersed. (A) Microcolony dispersal was examined in wild-type (WT) and Δ*pptB* bacteria (1 × 10^7^ CFU/ml) upon the addition of 10 mM l-lactate. A black horizontal line represents the 3-h time point of induction. The bars represent the means of three independent experiments. Error bars represent the standard deviations. ns, nonsignificant. (B) Microscopy images showing wild-type (WT) and Δ*pptB* bacteria upon addition of 10 mM l-lactate. DMEM was added as a control. Representative images are shown for 15, 50, 60, and 90 min after the addition of lactate. Scale bar, 20 μm. White arrows mark examples of microcolonies reformed after dispersal.

Upregulation of *pptB* upon host-cell contact has previously been shown to hinder Tfp interactions that lead to microcolony dispersal (6). Although mRNA level of *pptB* indicates that it is not involved in lactate-induced dispersal (8), we wanted to confirm this by using a Δ*pptB* mutant. We observed that deletion of *pptB* in FAM20 resulted in increased microcolony formation as described previously (6; data not shown). No difference in the timing of microcolony dispersal was detected compared to the wild type (Fig. 3A). Shortly after dispersal of the Δ*pptB* mutant, we observed reformation of microcolonies, indicating that PptB might be important for bacteria to remain in a planktonic state (Fig. 3B).

**FIG 4:**
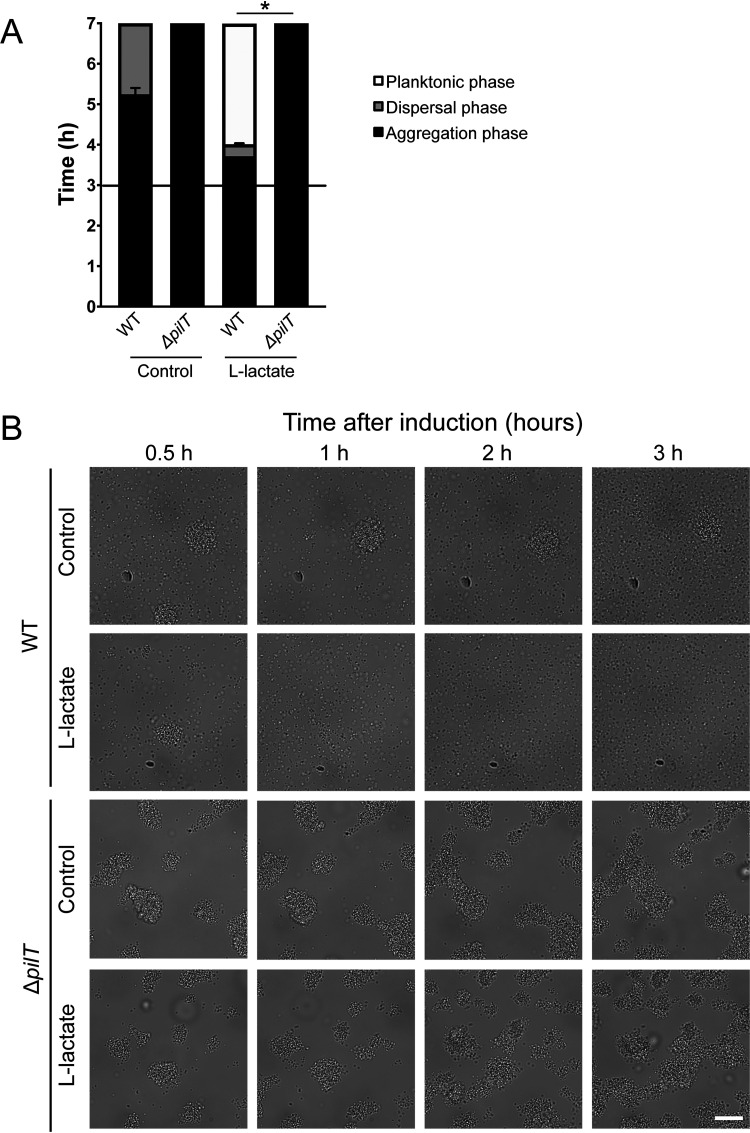
Lactate-induced dispersal is dependent on PilT retraction. (A) Microcolony dispersal was examined in wild-type (WT) and Δ*pilT* bacteria (1 × 10^7^ CFU/ml) upon the addition of 10 mM l-lactate. A black horizontal line represents the 3-h time point of induction. The bars represent the means of three independent experiments. Error bars represent the standard deviations. *, *P* < 0.05. (B) Microscopy images showing wild-type (WT) and Δ*pilT* bacteria (1 × 10^7^ CFU/ml) upon the addition of 10 mM l-lactate. Representative images are shown for 0.5, 1 h, 2. and 3 h. Scale bar, 20 μm.

**FIG 5:**
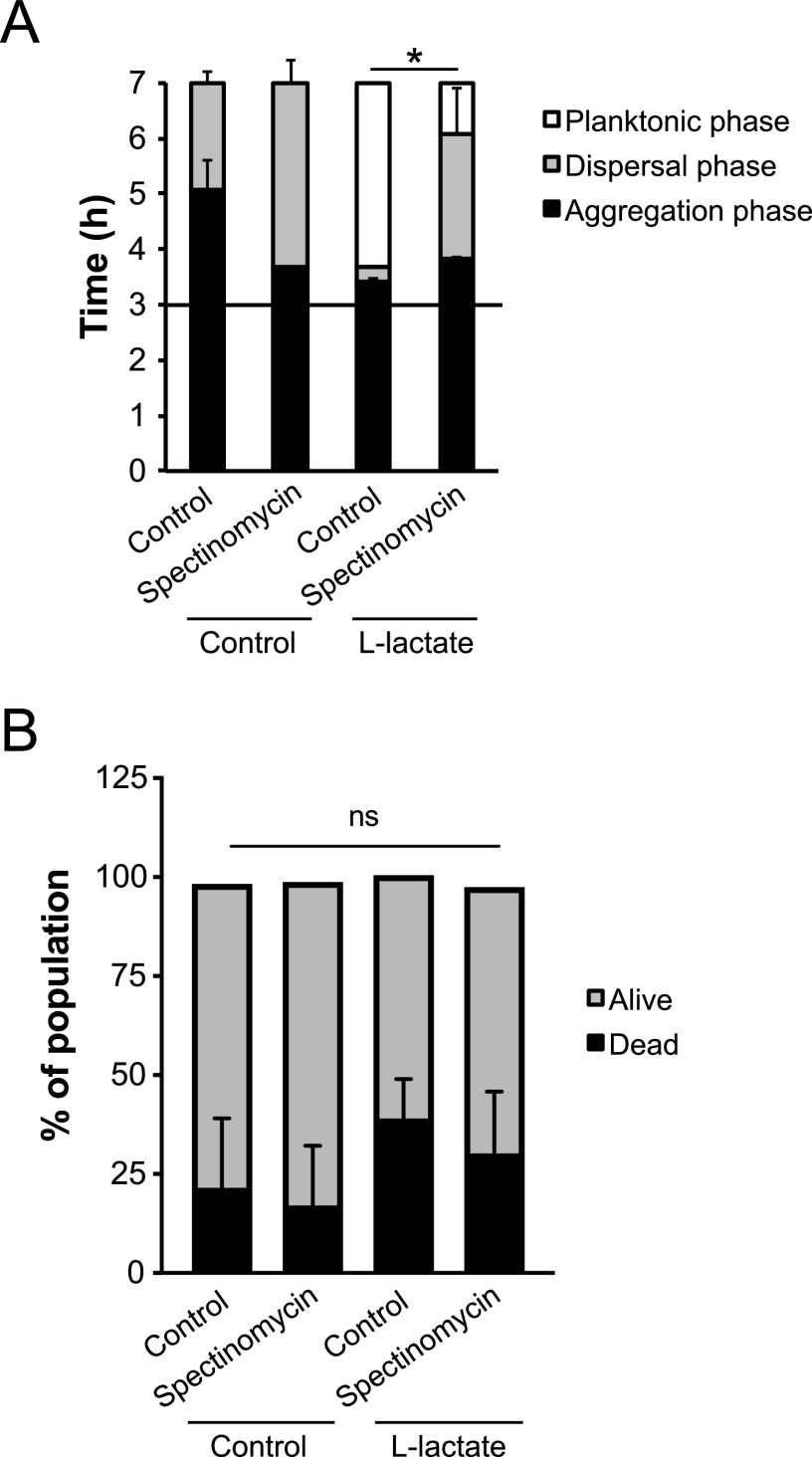
Effect of spectinomycin on microcolony dispersal. (A) Bacteria were resuspended to 10^7^ CFU/ml in DMEM containing 1% FBS and allowed to form aggregates for 2.5 h. The antibiotic spectinomycin was added at a final concentration of 100 μg/ml 30 min before the induction assay. After 3 h of incubation, lactate was added to final concentrations of 2 mM at a 1:1 ratio. DMEM was added as a control. The bars represent the means of two independent experiments. Error bars represent the standard deviations. *, *P* < 0.05. (B) Live-dead assays were performed to verify that bacteria were not killed by spectinomycin. Bacteria were stained in a live/dead assay at 60 min postinduction and analyzed by flow cytometry. Experiment was performed three times in duplicates. ns, nonsignificant.

Previous studies have shown that a loss of *pilT* in meningococci results in a hyperpiliated phenotype unable to retract the Tfp (32). Deletion of *pilT* also inhibits microcolony dispersal and intimate adhesion to host cells. To demonstrate that lactate-induced dispersal in liquid cultures was dependent on PilT-mediated retraction, we used a Δ*pilT* mutant in induction assays. The addition of lactate did not induce dispersal in the Δ*pilT* mutant, which remained in aggregates throughout the time-lapse experiment (Fig. 4). This suggests that PilT-mediated retraction rather than PTM, similar to phosphoglycerol modification by PptB, mediates lactate-induced dispersal in FAM20. Since Tfp retraction requires protein synthesis (33), we treated meningococci with spectinomycin 30 min before the addition of lactate. Spectinomycin treatment selectively inhibits protein synthesis, and the treatment delayed the dispersal, suggesting that protein synthesis was required (Fig. 5A). Since antibiotics frequently result in bacterial death, we performed a live-dead staining using flow cytometry. Treatment with spectinomycin did not increase bacterial death (Fig. 5B), suggesting that the phenotype was not due to a substantial proportion of the cells being dead compared to the untreated control. These results suggest that microcolony dispersal induced by lactate is dependent on PilT-mediated retraction and protein synthesis. PptB modification is not required for synchronized dispersal but is necessary for meningococci to remain dispersed.

### Temperature affects *N. meningitidis* dispersal and reformation of microcolonies.

**FIG 6:**
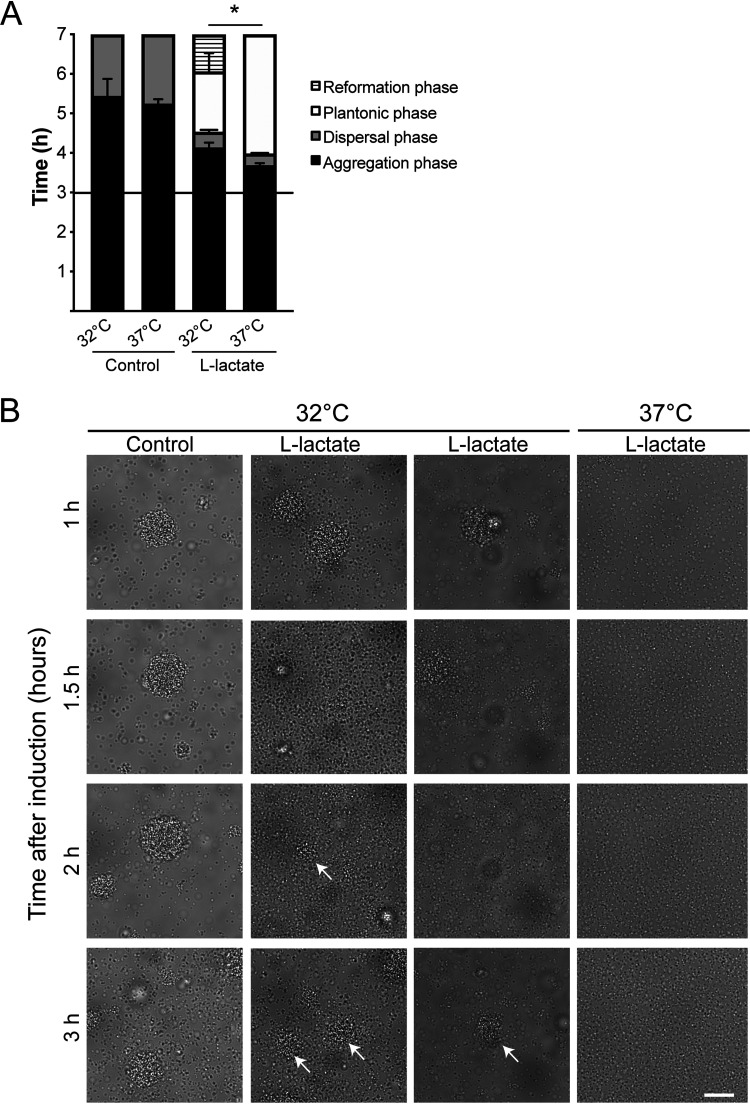
Reduced environmental temperature promotes reformation of microcolonies after dispersal. (A) Induction assays comparing microcolony dispersal of the wild-type (WT) at 32°C and 37°C. Bacteria were resuspended to 10^7^ CFU/ml in DMEM containing 1% FBS and allowed to form aggregates for 3 h. Lactate was added to final concentrations of 10 mM at a 1:1 ratio, and microcolony dispersal was followed by live-cell imaging. A black horizontal line represents the 3-h time point of induction. DMEM was added as a control. The bars represent the means of three independent experiments. Error bars represent the standard deviations. *, *P* < 0.05. (B) Microscopy images showing microcolony dispersal at 32°C compared to 37°C. Microcolonies had been allowed to form for 3 h before the addition of 10 mM lactate. For incubation at 32°C, two representative examples are shown for 1, 1.5, 2, and 3 h after induction with l-lactate. DMEM was added as a control. A representative example at 37°C is also shown. Experiment was performed three times in duplicates. Scale bar, 20 μm. White arrows mark examples of microcolonies reformed after dispersal.

Reduced temperature has been previously shown to affect protein expression of several virulence factors that enhance microcolony formation (27). Since the temperature in the nasopharynx is approximately 33°C ± 2°C (25), we were interested in examining the timing of dispersal at a lower temperature. We performed induction assays as previously described; bacteria were incubated at 32°C for 3 h, 10 mM lactate was added to the microcolonies, and timing of dispersal was examined. At 37°C, the synchronized dispersal phase occurred consistently at the same time, 30 min after lactate addition, and was fully dispersed within 1 h (Fig. 6A, 37°C l-lactate). In contrast, at 32°C, bacteria started to disperse around 1 h postinduction and then reformed microcolonies (Fig. 6A). Two representative examples are shown in Fig. 6B (32°C l-lactate lanes), where the bacteria started to disperse after 1 h and after some time re-entered the aggregation phase. Similar results were found using 2 mM lactate (data not shown). Although bacteria formed aggregates at 32°C and responded to lactate, the dispersal was delayed and microcolonies reformed again after 2 to 3 h. These results indicate that not only the lactate concentrations but also the environmental temperature can influence microcolony dispersal in meningococci.

## DISCUSSION

Understanding the mechanism behind microcolony dispersal is a major challenge in the field of meningococcal pathogenesis. Recently, we discovered that host-derived lactate induced the dispersal of meningococcal microcolonies (8). The answer to the question of how microcolonies respond to increased lactate concentration in the environment could help us to further understand disease progression. In this study, we focused on further characterization of factors contributing to lactate-induced dispersion. We showed that microcolony dispersal depended on bacterial concentration, although the QS-system AI-2 was not involved. Deletion of the response regulators of the TCS NarP/NarQ, PilR/PilS, NtrY/NtrX, and MisR/MisS had no effect on dispersal. Microcolony dispersal was dependent on *pilT-*mediated retraction and protein synthesis. Our results showed that the activity of PptB was not responsible for lactate-induced dispersal, but it prevented reaggregation. Finally, we suggest that environmental temperature can regulate microcolony formation and dispersal.

We found that dispersal in the presence of lactate was dependent on bacterial density. Little is known about N. meningitidis QS; the bacteria are known to produce functional QS signaling molecule AI-2, and its production is dependent on the *luxS* gene (12). The effect of QS on meningococcal microcolony dispersal has not been explored yet. Thus far, AI-2 is the only characterized QS system in *Neisseria*. We observed no differences in the timing of meningococcal microcolony dispersal upon the deletion of *luxS*. This finding indicates that AI-2-mediated QS does not affect lactate-induced microcolony dispersal. However, QS is involved in biofilm dispersal in several other pathogens, including Pseudomonas aeruginosa, Staphylococcus aureus, and Vibrio cholerae (reviewed in reference 34).

TCS are commonly used by bacterial pathogens to sense metabolite levels and other environmental stimuli such as QS molecules, temperature, pH, antimicrobials, and oxygen levels (35). Since TCS enable bacteria to sense and respond to extracellular signals, we sought to determine whether they might play a role in the lactate-induced dispersal. Lactate has previously been demonstrated to affect the TCS activity. In Escherichia coli, d-lactate can boost activated ArcB QS-regulator (36, 37). Lactate can also activate the three-component system LrbS/LrbA/LrbR in Shewanella putrefaciens. The LrbS/LrbA/LrbR system contains two response regulators that negatively regulate biofilm formation upon activation (38). In addition, pyruvate, the downstream metabolite of lactate, activates three TCS in S. aureus, thereby enhancing its virulence (39). However, inactivation of the meningococcal TCS NarP/NarQ, PilR/PilS, and NtrY/NtrX of MisR/MisS did not prevent the lactate-induced dispersal of microcolonies.

*misR* and *misS* deletion mutants have previously been successfully constructed in N. meningitidis serogroups A, B, and C, although not in strain FAM20. Deletion of the genes resulted in phenotypes that were severely growth deficient and sometimes unable to grow at low magnesium levels (15, 18, 31, 40, 41). We were not able to generate a *misR* deletion mutant in FAM20, and differences between strains might be the reason for this. Expression of *misR* and *misS* has been shown to be upregulated upon contact with host cells, and the TCS is proposed to be involved in adaptation to growth on cells (18).

When we examined the *ΔntrX* mutant in FAM20 by live-cell microscopy, we observed an unusual phenotype suggesting that the mutant was deficient in bacterial cell division. Deletion mutant of *ntrX*, encoding the regulator of the TCS NtrY/NtrX in Neisseria gonorrhoeae has been described previously. Expression of genes involved in aerobic and anaerobic respiration was downregulated, which resulted in defects in both biofilm formation and survival within human epithelial cells. However, the *ntrX* mutant was not growth deficient under both aerobic and microaerobic conditions (22). It is not known whether the *ntrX* mutation affects respiration in meningococci and whether this results in the observed deficiency in cell proliferation.

It is known that the absence of PilF ATPase activity and the presence of PilT ATPase activity in N. meningitidis leads to a rapid disappearance of pili from the bacterial surface and the dispersal of aggregates (42). Therefore, we examined whether the lactate-induced microcolony dispersal was a result of alterations in Tfp biogenesis. However, there were no detectable differences in the transcripts of Tfp-related genes during the process of meningococcal microcolony dispersal.

PptB modifies PilE posttranslationally by conjugating a phosphoglycerol moiety to the serine residue 93, which has a negative impact on pilus-pilus interactions and facilitates the detachment of bacteria from microcolonies (6). We observed that the Δ*pptB* mutant responded to l-lactate the same way as the wild type, showing that phosphoglycerol modification of PilE was not required for the induction of rapid and synchronized dispersal. We noted, however, that a loss of PptB activity resulted in subsequent reformation of microcolonies upon dispersal. The expression of *pptB* has been reported to be induced upon contact with host cells controlled by the CREN regulon (17). However, no increase in *pptB* expression was observed when microcolony dispersal was induced by lactate in liquid cultures (8). The PTM patterns can vary greatly between the different classes (6, 43). The expression level of *pptB* did not change over time but remained stable up to 60 min after lactate induction. It needs to be taken into account that the serogroup C strain 8013 used by Chamot-Rooke et al. expresses class I Tfp, whereas the FAM20 used in this study expresses class II Tfp.

We did not detect any detachment of bacteria from the microcolonies formed by Δ*pilT* mutant throughout the time-lapse (Fig. 4). This suggests that the process is dependent on Tfp retraction rather than PTM that block Tfp bundle formation. Since the mRNA and protein level of PilE did not increase upon lactate-induced dispersal (8), it also indicates that the retraction of the Tfp is needed, not the loss of piliation.

The filamentous prophage MDAΦ, expressed by meningococcal disease isolates, plays a role in colonization of the nasopharynx. MDAΦ structure resembles the Tfp and is also assembled through the PilQ porin (44, 45). Billie et al. have shown that bacteria in direct contact with epithelial cells mainly express Tfp mediating adhesion, while bacteria not in direct contact express the MDAΦ form bundles and mediate bacterial interaction (45). The strain used in our experiments, FAM20, is known to possess the MDA island encoding the filamentous prophage (44). The role of MDAΦ in microcolony dispersal has not been established. However, since lactate-induced dispersal has been reported in N. gonorrhoeae, which does not contain the genetic island (46), MDAΦ is unlikely to play a role.

It is becoming more evident that meningococcal virulence factors are thermally regulated. Our results suggest that microcolony dispersal is under thermal control. Although dispersal occurred at 32°C, the timing was not synchronized, and we observed aggregate formation shortly after the dispersal phase ended. Lappann et al. have shown that the protein levels of surface factors contributing to a carrier state are induced at 32°C, resembling the environmental temperature in the upper respiratory tract. Major changes were observed in levels of neisserial heparin binding antigen (NhbA), NMB1030, and adhesion complex protein (ACP), involved in meningococcal aggregation, adhesion, and biofilm formation (27). It is tempting to hypothesize that upregulation of these proteins might play a role in the subsequent reformation of the microcolonies upon dispersal.

**FIG 7:**
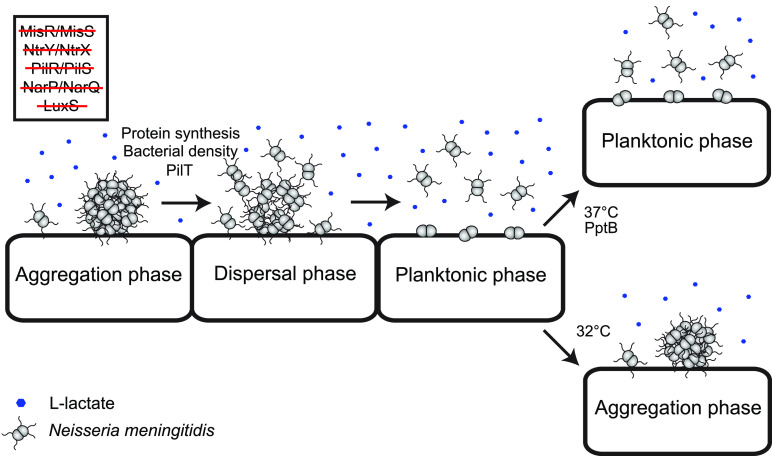
Schematic model of the proposed sequence of events during lactate-induced microcolony dispersal. For meningococci to detach from microcolonies in the presence of lactate, PilT-mediated retraction, protein synthesis, and a certain bacterial density are required. To remain in planktonic phase, meningococci depend on PTM by PptB and optimal environmental temperature. At lower temperatures, meningococci reform microcolonies. Finally, the data suggest that AI-2, PilR/PilS, NarP/NarQ, NtrX/NtrY, and MisR/MisS are not involved.

Collectively, our results suggest a hypothetical model (Fig. 7). Meningococcal microcolony dispersal is dependent on the bacterial concentration but not on the QS system AI-2 and the TCS. N. meningitidis depends on PilT-mediated retraction and protein synthesis for lactate-induced microcolony dispersal. To remain in a dispersed state, meningococci require the PptB activity and an optimal environmental temperature. These findings contribute to the understanding of the complex actions of N. meningitidis during microcolony dispersal. Further research may provide new targets for disease prevention.

## MATERIALS AND METHODS

### Bacterial strains and growth conditions.

The Neisseria meningitidis serogroup C strain FAM20 and its isogenic *pilT* mutant have been described previously (47). The *misR* (also called *phoP*) deletion mutant has been described (31). Prior to experiments, the strains were grown for 16 to 18 h on GC agar supplemented with 1% Kellogg medium at 37°C in 5% CO_2_. Dulbecco modified Eagle medium (DMEM; Thermo Fisher Scientific) containing 1% heat-inactivated fetal bovine serum (FBS; Sigma-Aldrich) was used in all experiments. Kanamycin was used at 50 μg/ml for the selection of mutants.

### Construction of mutant strains.

All primers used for cloning are listed in Table 1. To construct deletion mutants, fusion PCRs were performed in two steps. First, upstream (UHS, primer pair number 1) and downstream (DHS, primer pair number 2) sequences of the genes of interest were amplified from chromosomal DNA. The DNA uptake sequence was incorporated into the primers if not present in the upstream and downstream sequences (Table 1). Antibiotic resistance cassettes were amplified by using primer pair number 3. Primers contained overlapping sequences; therefore, a fusion product of UHS_Resistance-cassette_DHS could be created from PCR products using separate fragments as the templates. The resulting product from the fusion PCR could be inserted into the genome of N. meningitidis FAM20 using homologous allelic replacement with spot transformation. High-Fidelity Phusion DNA polymerase (Thermo Fisher Scientific) was used for all PCRs. The FAM20 *ΔpptB* mutant was created using homologous allelic replacement after spot transformation with *ΔpptB* mutant chromosomal DNA (6). Mutants were sequenced (MWG Eurofins) to confirm the correct location and sequence.

**TABLE 1:** Primers used in the study

Primer	Sequence (5′–3′)^*a*^	Source or reference
luxS_UHS_fwd_1	CACCATAGCCGCATTGTGTGT	This study
luxS_UHS_rev_1	GGTCACTAATACGGTCATGGTTTTCGCCACG	This study
luxS_DHS_fwd_2	AGTTTTTCTAACAATGCGGCACTTATCAAATG	This study
luxS_DHS_rev_2	AAAATAGGGAAGGACGATGC	This study
luxS_Kan_fwd_3	ACCATGACCGTATTAGTGACCTGTAGAATTCGAG	This study
luxS_Kan_rev_3	TGCCGCATTGTTAGAAAAACTCATCGAGCATCAAATG	This study
narP_UHS_fwd_1	GGCGGACGCTTTACCATGA	This study
narP_UHS_rev_1	GGTCACTAATACTAATCATTTTGATGCCCGAGAGG	This study
narP_DHS_fwd_2	AGTTTTTCTAACACCGTCAAAGTCCACGTTC	This study
narP_DHS_rev_2	*TTCAGACGGCAT*GGGATTCGGAATGGAACATTTTAC	This study
narP_Kan_fwd_3	CAAAATGATTAGTATTAGTGACCTGTAGAATTCGAG	This study
narP_Kan_rev_3	CTTTGACGGTGTTAGAAAAACTCATCGAGCATCAAATG	This study
pilR_UHS_fwd_1	AAACAGCCCGGCTCGATTTC	This study
pilR_UHS_rev_1	TCTACAGGTCACTAATACCCATTTTCATCAGGGTCATTTCC	This study
pilR_DHS_fwd_2	CGATGAGTTTTTCTAAACCCCGATACCATGCAGATA	This study
pilR_DHS_rev_2	TGTTTTGTGTCCGTTCGG	This study
pilR_Kan_fwd_3	CCTGATGAAAATGGGTATTAGTGACCTGTAGAATTCGAGCT	This study
pilR_Kan_rev_3	TGGTATCGGGGTTTAGAAAAACTCATCGAGCATCAAATG	This study
ntrY_UHS_fwd_1	CAAGGAAATGCTGCACAACG	This study
ntrY_UHS_rev_1	TCTACAGGTCACTAATACTAACCTTCGTCCTGCAGGATTT	This study
ntrY_DHS_fwd_2	CGATGAGTTTTTCTAAAATGTGGCGGAAAGCCAAAA	This study
ntrY_DHS_rev_2	*TTCAGACGGCAT*TCTGAAAACAAAAGCCCGCA	This study
ntrY_Kan_fwd_3	GCAGGACGAAGGTTAGTATTAGTGACCTGTAGAATTCGAGC	This study
ntrY_Kan_rev_3	TTTCCGCCACATTTTAGAAAAACTCATCGAGCATCAAATG	This study
rpsJ_fwd	TTGGAAATCCGCACCCACTT	48
rpsJ_rev	TACATCAACACCGGCCGACAAA	48
pilF_fwd	TCCACCCTGCACACCAATAAT	This study
pilF_rev	GAACACAGCCTGCGTAAAAGAC	This study
pilT_fwd	GTCGACCGTATCGTGGACGTATT	49
pilT_rev	TTCAGCAGGTTTTGGGAGATGAC	49
pilE_fwd	TATTCCGACAACGGCACATTCCC	48
pilE_rev	CCTTCAACCTTAACCGATGCCA	48
pilD_fwd	TCGGTATGGGCAACGGAGAT	This study
pilD_rev	CAGCGAGGAAACAAAAATCAGC	This study
pilQ_fwd	GTCAAAATCAACAAGGACTCGC	This study
pilQ_rev	TTGTCTTCTTCATAAATACCGCC	This study
pptB_fwd	AAGGCGTGGAAGTCATCATC	8
pptB_rev	TGTTTGAGGTAGGTAGCGGAAGGT	8

a The DNA uptake sequence is market in italics. Overlapping sequences for fusion PCR are underlined.

### Live-cell imaging.

N. meningitidis FAM20 and its isogenic mutants were resuspended in prewarmed DMEM containing 1% FBS, filtered through 5-μm filters to break existing aggregates, and diluted to preferred bacterial concentrations (2 × 10^7^, 1 × 10^7^, 8 × 10^6^, 6 × 10^6^, 4 × 10^6^, and 2 × 10^6^) in the presence of 2 mM l-lactate (L7022; Sigma-Aldrich). Microcolony dispersal was observed using time-lapse microscopy (Axiovert Z1; Zeiss). Three positions were chosen per well, and images were acquired every 10 min for 6 h using a 40× objective. The images were independently evaluated by visual inspection. The aggregation phase was defined as the time from the start of incubation until dispersal of bacteria starts (i.e., when the microcolonies reached maximal size and started to decrease size). The dispersal phase was defined as the time from initiation of bacterial dispersal until microcolonies were no longer visible in the images. The planktonic phase was described as dispersed single bacteria. For wild-type strain and 5 mM lactate, the dispersal was rapid and obvious and occurred from one image to another, i.e., within a 10-min time frame.

### Induction assay.

The induction assays have been described previously (8). Briefly, bacteria were resuspended in prewarmed DMEM containing 1% FBS, filtered through 5-μm filters, and diluted to 10^7^ CFU/ml. The bacterial solutions were transferred to 24-well glass-bottom plates (MatTek) and allowed to grow and form microcolonies for 3 h at 32°C or 37°C in a 5% CO_2_ environment in a live-cell observer (Axiovert Z1; Zeiss). Prewarmed DMEM supplemented with sodium l-lactate (L7022; Sigma-Aldrich) was added to microcolonies at a 1:1 ratio at final concentrations of 10 mM. During treatment with spectinomycin, the antibiotic was added at a final concentration of 100 μg/ml 30 min before the induction. Three positions were chosen per well, and images were acquired every 5 to 10 min for 4 or 5 h using a 40× objective. The aggregation phase is described as the start of incubation until the dispersal of bacteria starts. The dispersal phase is described as the initiation of bacterial dispersal until no aggregates are observed. The planktonic phase is described as dispersed single bacteria.

### Live-dead staining.

Bacteria were treated with 100 μg/ml of spectinomycin 30 min before induction with l-lactate. At 60 min postinduction, bacteria were stained with a Live/Dead BacLight bacterial viability kit (L7012; Invitrogen) according to the manufacturer’s protocol. The samples were analyzed by flow cytometry using an LSRFortessa (BD Biosciences) with a PI channel for dead cells and FITC for live cells. For each sample, 15,000 bacterial cells were counted, and data are presented as the percentages of dead or alive bacteria within this population. Data were analyzed with the FlowJo software.

### Quantitative real-time PCR analysis.

All of the primers used for quantitative real-time PCR analysis are listed in Table 1. Induction assays with l-lactate and control DMEM were performed on FAM20 wild type as indicated above in 1-ml volumes in 2.0-ml Eppendorf tubes. Samples were collected at 1, 5, 10, 20, 30, and 60 min immediately after the addition of l-lactate or DMEM and treated with RNAprotect bacterial reagent (Qiagen). Total RNA was purified using an RNeasy plus minikit (Qiagen) according to the manufacturer’s protocol. Reverse transcription was performed with random hexamers using a SuperScript VILO Master Mix (Thermo Fisher). The resulting cDNA was amplified using LightCycler 480 SYBR green I Master mix (Roche) in a LightCycler 480 real-time PCR system. The PCR program was adapted from the manufacturer using an annealing temperature of 55°C. The 30S ribosomal protein *rpsJ* was used as a reference gene. The target mRNA levels in the samples were normalized to the reference gene and then compared to the value of the noninduced DMEM control sample.

### Statistical analysis.

For statistical analysis in induction assays, the differences between the lengths of the dispersal phase were analyzed. In Excel, two-tailed and unpaired Student *t* tests were used to compare differences between two groups. In GraphPad Prism (version 5), analysis of variance with Bonferroni’s *post hoc* test was used to compare differences between multiple groups. *P* values below 0.05 were accepted as statistically significant.
