# Supplementary material for: Lactate-Induced Dispersal of Neisseria meningitidis Microcolonies Is Mediated by Changes in Cell Density and Pilus Retraction and Is Influenced by Temperature Change
Source: Infect Immun. 2021 Sep 16;89(10):e00296-21. doi: 10.1128/IAI.00296-21 (PMC8445170; doi:10.1128/IAI.00296-21)

Supplementary file

Fig. S1. Time course expression of genes associated with Tfp biogenesis in *N. meningitidis* upon lactate induction. Samples were taken 1, 5, 10, 20, 30, 60 min after addition of lactate. Target mRNA levels were normalized to the housekeeping gene coding for the 30S ribosomal protein RpsJ. Gene transcripts levels in the controls were set to a value of 1. Data represent the mean ± SD of three independent experiments.

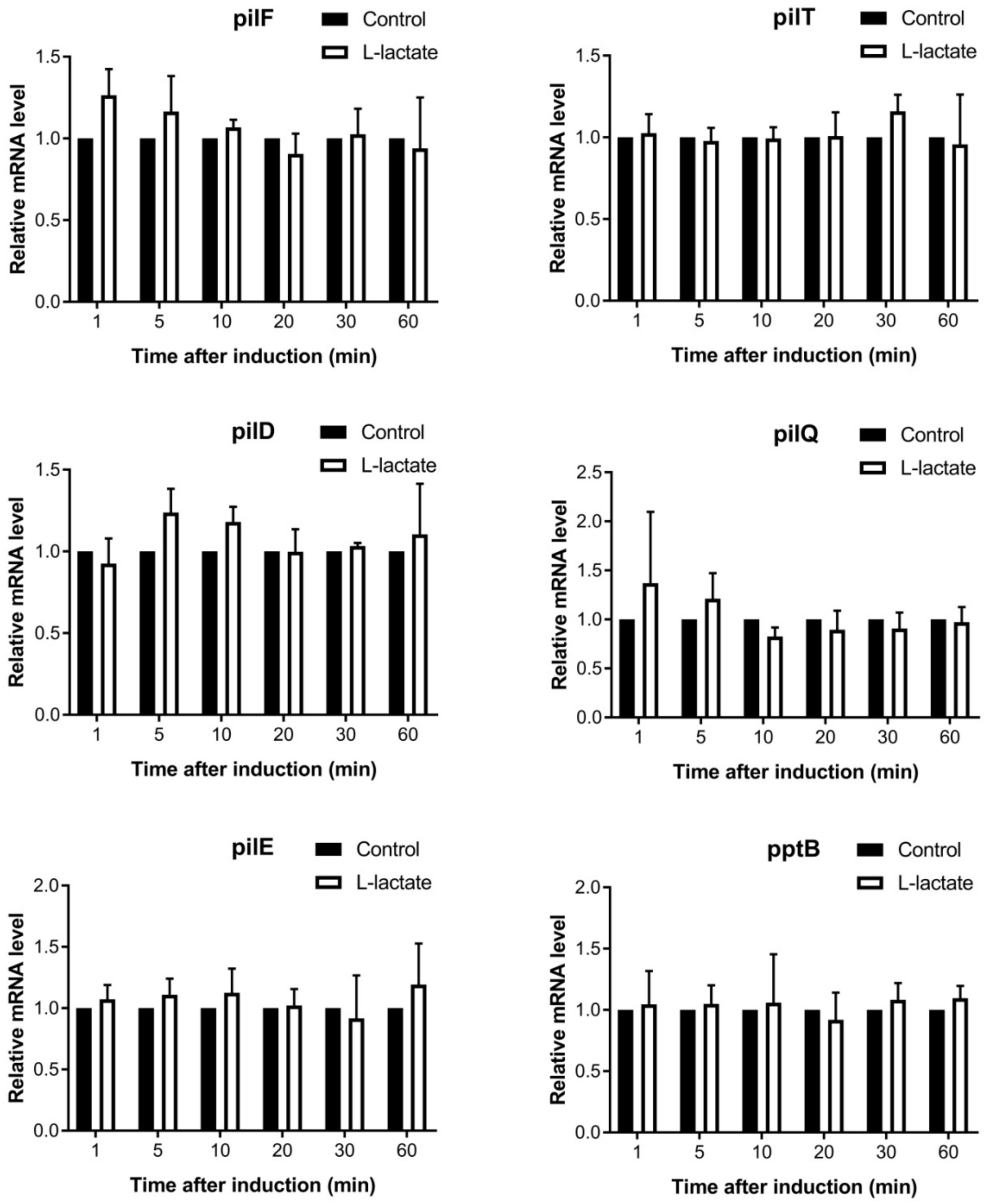

Supplement: Supplemental file 1 — Supplemental material. Download IAI.00296-21-s0001.pdf, PDF file, 0.3 MB [file iai.00296-21-s0001.pdf]
